# CCP1, a Tubulin Deglutamylase, Increases Survival of Rodent Spinal Cord Neurons following Glutamate-Induced Excitotoxicity

**DOI:** 10.1523/ENEURO.0431-20.2021

**Published:** 2021-03-31

**Authors:** Yasmin H. Ramadan, Amanda Gu, Nicole Ross, Sara A. McEwan, Maureen M. Barr, Bonnie L. Firestein, Robert O’Hagan

**Affiliations:** 1 Department of Genetics, Human Genetics Institute of New Jersey, Rutgers, The State University of New Jersey, Piscataway, NJ 08854; 2 Biology Department, Montclair State University, Montclair, NJ 07043; 3 Department of Cell Biology and Neuroscience, Rutgers, The State University of New Jersey, Piscataway, NJ 08854

**Keywords:** cilia, excitotoxicity, neuronal injury, neuroprotection, polyglutamylation, spinal cord

## Abstract

Microtubules (MTs) are cytoskeletal elements that provide structural support and act as roadways for intracellular transport in cells. MTs are also needed for neurons to extend and maintain long axons and dendrites that establish connectivity to transmit information through the nervous system. Therefore, in neurons, the ability to independently regulate cytoskeletal stability and MT-based transport in different cellular compartments is essential. Posttranslational modification of MTs is one mechanism by which neurons regulate the cytoskeleton. The carboxypeptidase CCP1 negatively regulates posttranslational polyglutamylation of MTs. In mammals, loss of CCP1, and the resulting hyperglutamylation of MTs, causes neurodegeneration. It has also long been known that CCP1 expression is activated by neuronal injury; however, whether CCP1 plays a neuroprotective role after injury is unknown. Using shRNA-mediated knock-down of CCP1 in embryonic rat spinal cord cultures, we demonstrate that CCP1 protects spinal cord neurons from excitotoxic death. Unexpectedly, excitotoxic injury reduced CCP1 expression in our system. We previously demonstrated that the CCP1 homolog in *Caenorhabditis elegans* is important for maintenance of neuronal cilia. Although cilia enhance neuronal survival in some contexts, it is not yet clear whether CCP1 maintains cilia in mammalian spinal cord neurons. We found that knock-down of CCP1 did not result in loss or shortening of cilia in cultured spinal cord neurons, suggesting that its effect on survival of excitotoxicity is independent of cilia. Our results support the idea that enzyme regulators of MT polyglutamylation might be therapeutically targeted to prevent excitotoxic death after spinal cord injuries.

## Significance Statement

Combining an *in vitro* model of the secondary phase of spinal cord injury with shRNA knock-down, we demonstrate that the deglutamylase CCP1 protects neurons from excitotoxic death. Excitotoxicity plays a role in the secondary phase of neuronal injuries, contributing to neurodegeneration. CCP1 function was previously known to be associated with cilia. We provide the first demonstration (to our knowledge) that spinal cord interneurons are ciliated. However, our data suggest that neuroprotection by CCP1 may be independent of cilia in spinal neurons. Our work supports the idea that targeting enzymes that modify tubulins, such as glutamylases and deglutamylases, might be an avenue of treatment for nervous system injuries.

## Introduction

The development, function, and survival of neurons rely heavily on the function of the microtubule (MT) cytoskeleton (Witte and Bradke, 2008; Varidaki et al., 2018). MTs are hollow cylinders formed by polymerization of α and β tubulin subunits (Pellegrini et al., 2017). Tubulins are highly conserved, differing little in sequence and structure (Gadadhar et al., 2017); however, MTs in different neuronal compartments, such as axons, dendrites, or growth cones, display differences in function and dynamics (Witte and Bradke, 2008). The tubulin code model proposes that in addition to heterogeneity of tubulin isotype composition, posttranslational modification of tubulins can specialize the stability, form, and function of MTs (Verhey and Gaertig, 2007; Gadadhar et al., 2017). Tubulin code modifications are proposed to endow specific MTs with particular properties to play essential roles in the function of axons and dendrites as well as directional trafficking, plasticity, and survival (Verhey and Gaertig, 2007; Gadadhar et al., 2017). Glutamylation, one component of the tubulin code, consists of side-chains of the amino acid glutamate that are posttranslationally added to the carboxy terminal tails of tubulins when assembled into MTs (Eddé et al., 1990; Bre et al., 1994). Glutamylation often decorates MTs in both neurons and cilia (Gadadhar et al., 2017).

The primary cilium, a non-motile sensory organelle that protrudes from most non-dividing cells in the human body, is also a region of MT specialization (Werner et al., 2017). Conserved over eukaryotic evolution from algae to vertebrates, the architecture of cilia consists of a MT cytoskeleton, called the axoneme, in which a ring of nine MT doublets extends along cilia immediately beneath the membrane (Werner et al., 2017). Vertebrate cilia function in processes such as kidney function, olfaction, vision, and development of left-right asymmetry (Youn and Han, 2018). Cilia also play a role in nervous system development because of their function as an essential hub for signaling pathways (Lee and Gleeson, 2011; Bay and Caspary, 2012).

Recently, primary cilia that protrude from mammalian neurons have been proposed to play an essential role in maintaining neuronal viability and connectivity (Bowie and Goetz, 2020). Loss of neuronal cilia in the mammalian brain causes neurodegeneration and synapse loss and may be a primary cause of motor coordination defects in spinal cerebellar ataxia (SCA; Bowie and Goetz, 2020). Although neuronal injury can cause ablation of cilia, cilia exert a neuroprotective effect (Choi et al., 2019).

Glutamylation regulates the structure and function of cilia (Ikegami and Setou, 2010; O’Hagan et al., 2011, 2017). M14D carboxypeptidases, such as CCP1 (Rodriguez de la Vega et al., 2007), remove or reduce the length of glutamate side-chains (Rogowski et al., 2010). When deglutamylase function is lost, hyperglutamylation affects ciliary motor transport and causes degeneration of some types of neuronal sensory cilia in *Caenorhabditis elegans* (O’Hagan et al., 2011; Power et al., 2020) . In mice, loss of CCP1 leads to the degeneration of retinal photoreceptors and sperm defects (Fernandez-Gonzalez et al., 2002). These phenotypes are reminiscent of the symptoms of diseases caused by ciliary dysfunction or “ciliopathies” (Mitchison and Valente, 2017).

Glutamylation also occurs on non-ciliary neuronal MTs (Fukushima et al., 2009). Loss of CCP1 perturbs neuronal transport in mice and humans (Fernandez-Gonzalez et al., 2002; Ikegami et al., 2007; Magiera et al., 2018) and leads to infantile hereditary neurodegeneration and cerebellar atrophy in humans (Shashi et al., 2018). In mammals, expression of CCP1 deglutamylase is upregulated in response to transection or crush injury of the sciatic nerve, suggesting that its function may be required for neuroregeneration (Harris et al., 2000). Loss of deglutamylase activity diminishes regrowth of laser-severed neurons in *C. elegans*, supporting a possible conserved role in neuroregeneration (Ghosh-Roy et al., 2012).

Questions about the function of CCP1 remain unanswered. Is CCP1 neuroprotective or does it play a pathologic role after neuronal injury? Does CCP1 expression increase in injured neurons of the CNS as it does in the sciatic nerve in the peripheral nervous system (PNS)? Is the role of CCP1 in ciliary maintenance also important in injured neurons?

Here, using an *in vitro* model of the secondary phase of spinal cord injury (Du et al., 2007), we find that knock-down of CCP1 decreases the survival of spinal cord neurons subjected to excitotoxic glutamate treatment. In contrast to the reported upregulation of CCP1 in response to injury of the sciatic nerve, our analysis showed that glutamate-induced excitotoxic injury reduces CCP1 expression in spinal cord neurons. However, shRNA CCP1 knock-down in cells subjected to excitotoxic glutamate did not reduce CCP1 expression to lower levels than excitotoxic glutamate alone.

We also used shRNA to knock down CCP1 expression to test whether CCP1 activity affects neuronal cilia in embryonic rat spinal cord cultures. Using immunofluorescence-based detection of the ciliary marker ARL13B, we found that a primary cilium protrudes from the majority of neurons in our spinal cord cultures. Unexpectedly, CCP1 knock-down did not decrease the percentage of ciliated neurons or ciliary length.

Our data suggest that CCP1 activity is protective for neurons and support the idea that targeting regulators of MT glutamylation may offer a new option for treatment of excitotoxic damage resulting from nervous system injury.

## Materials and Methods

All animal experiments were conducted in accordance with the National Institutes of Health (NIH) *Guide for the Care and Use of Laboratory Animals* (NIH Publication No. 8023, revised 1978).

### Spinal cord cultures

Our method was similar to that used by Du et al. (2007). Briefly, spinal cords were dissected from Sprague Dawley rat embryos at gestational day 15 [embryonic day (E)15] by removing meninges and attached dorsal root ganglia. Cords were then gently triturated to dissociate a mixture of neurons, astrocytes, and microglia from the tissue. Cells were plated at a density of ∼523 cells/mm^2^, or 100,000 cells per well, in a 24-well plate containing coverslips coated with 0.01% solution of poly-d-lysine (Sigma) dissolved in 0.1 m borate buffer (sodium tetraborate, boric acid) for cell adhesion. Mixed cultures were grown in DMEM (Invitrogen) supplemented with 10% horse serum (Invitrogen) for 7 d before glutamate treatment.

### Lentiviral production and infection

Lentiviruses were produced as previously described (Patel et al., 2019). In summary, HEK293T cells, plated at a density of 6.5 × 10^6^ cells/T75 flask were grown for 1 d and then transfected on day *in vitro* (DIV)2 with lentiviral plasmids (VectorBuilder) carrying scrambled shRNA (target sequence: CCTAAGGTTAAGTCGCCCTCG; vector ID: VB170329-1128paq), CCP1 knock-down shRNA (target sequence: CCACTTCCAGTTGCCAATTAT; vector ID: VB171011-1192pab), or CCP1 cDNA (RGD ID: 1306307; AGTPBP1 also known as CCP1) and GFP or RFP fluorescent markers. VSV and PAX2 packaging vectors and Lipofectamine 2000 (Invitrogen) were used according to the manufacturer’s protocol to mediate transfection. After 3 d, the media were collected and centrifuged at 1500 × *g* for 5 min to pellet dead cells and debris. The supernatant was then collected and incubated with PEG-it (System Biosciences) in a 5× solution at 4C for 2 d to precipitate the viruses. On day 7, the solution was centrifuged at 1500 × *g* for 30 min at 4C to pellet the viruses. The supernatant was removed and discarded, and the virus pellet was resuspended in 150 μl of sterile 1× PBS and frozen (at −80°C) in 5 μl aliquots until use.

All spinal cord culture infections were performed on DIV2 at a 1:5000 dilution by replacing one-fourth of the medium in the well with a 1:1250 dilution of virus in fresh DMEM + 10% horse serum.

### Glutamate treatment

Glutamate-induced excitotoxicity was performed as previously described (Du et al., 2007). Glutamate (L-glutamic acid, Sigma Life Sciences) dilutions were made from a 1 mm stock solution dissolved in DMEM + 10% horse serum warmed to 37°C. During treatment on DIV7, medium was collected from each well and replaced with varying concentrations of glutamate-containing medium as described in results for 1 h at 37°C and 5% CO_2_. Conditioned medium (collected before treatment) was combined with an equal volume of fresh medium (1:1 solution) and used as recovery medium following glutamate treatment. Cells were allowed to recover for 24 h at 37°C and 5% CO_2_ before fixation.

### Immunocytochemistry

Cells were fixed by incubation in 4% paraformaldehyde in 1× PBS for 15 min. Following fixation, cells were washed three times with 1× PBS and incubated in a blocking solution (2% normal goat serum, 0.1% Triton X-100, 0.02% NaN_3_ diluted in 1× PBS) for 1 h. Cells were incubated in primary antibody solution (1:500) overnight (∼18 h), washed three times in 1× PBS, and incubated with appropriate secondary antibody solutions (1:1000) for 1 h the next day, followed by another three washes in 1× PBS. Coverslips were then removed and mounted on glass slides for imaging.

Anti-MT-associated protein 2 (MAP2) antibody (ThermoFisher) was used to identify neurons. Anti-GFAP (glial fibrillary acidic protein; Millipore, rabbit) was used to label astrocytes, and anti-IBA1 (ionized calcium binding adaptor molecule 1; Proteintech, mouse) was used to label microglia. Cilia were immunolabeled using a monoclonal anti-ARL13B antibody (Proteintech, mouse). PolyE antibody (Adipogen catalog #AG-25B-0030-C050), a polyclonal antibody which only binds polyglutamylated substrates (three or more glutamate residues; Rogowski et al., 2010), was used to visualize polyglutamylated MTs. Anti-choline acetyltransferase (ChAT; Millipore Sigma, goat) antibody was used to identify motorneurons. Alexa Fluor-conjugated secondary antibodies (488, 568, 647) were used to visualize cell-specific markers. Nuclei were labeled using NucBlue Live Ready Probes reagent (ThermoFisher), which was excited at 350 nm.

### Imaging and microscopy

Image Z-stacks were acquired with MetaMorph software (Molecular Devices) using a Zeiss Axioplan2 microscope with 10×, 63× (NA 1.4), and 100× (NA 1.4) oil-immersion objectives, equipped with a Hamamatsu C11440-42U ORCA-Flash4.0 LT Digital CMOS camera (Hamamatsu). Images were uploaded into FIJI/Image J 2.0 to create optical Z-stack projections, add scale bars, and adjust brightness/contrast. The cell counter plugin was used to count MAP2-immunopositive and GFP-positive cells. Images were then exported as PNG files for assembly into figures in Adobe Illustrator CS.

### Scoring neuronal survival

To assess neuronal survival following glutamate treatment, neurons identified by MAP2 immunostaining were counted from five 1.4 × 1.4 μm regions imaged from each coverslip using the 100× objective and analyzed as ratios of neurons remaining following glutamate treatment/neurons present in the absence of a glutamate treatment.

### Scoring neuronal ciliation and cilia length

Neurons were identified using anti-MAP2 immunofluorescence, and viral infection with shRNA vectors (scrambled or anti-CCP1) was scored by expression of GFP. For ciliation, the presence or absence of a cilium immunolabeled by ARL13B on the cell body of each neuron in 5 randomly chosen areas was scored and analyzed from Z-stacks of images taken using the 100× objective on the Zeiss Axioplan2 microscope. Z-stacks were uploaded into FIJI/ImageJ, Z-projected, and neuronal cilia were counted using the Cell Counter plugin.

For cilia length, only neurons with “horizontally-projecting” cilia (the entire length of the cilium was visible in a single focal plane of a Z-stack) were scored from images taken with the 100× objective on the Zeiss Axioplan2 microscope. Z-stacks were uploaded into FIJI/ImageJ, Z-projected, and measured for pixel length using the tracing tool. Pixels were converted to microns (at 100×, 0.6566 μm = 1 pixel) before graphing and statistical analysis.

### Western blot analysis

For Western blot assays, spinal cord cultures were grown at 1 million cells per well in a six-well plate and infected with viruses and/or treated with glutamate at similar concentrations as described above. On DIV8, cells were homogenized in RIPA buffer (50 mm Tris-HCl, pH 7.4, 150 mm NaCl, 0.5% deoxycholate, 1% NP-40, 0.1% SDS, and 1 mm EDTA, pH 7.4) by scraping the cells into the buffer and sonication. Protein concentrations were determined using the Pierce BCA protein assay kit (Thermo Scientific) according to the manufacturer’s protocol. Proteins (30 μg/lane) were resolved on a 10% SDS polyacrylamide gel and transferred to a polyvinylidene difluoride (PVDF) membrane. Membranes were blocked with 5% bovine serum albumin (BSA) in 1× TBST (20 mm Tris, pH 7.5, 150 mm NaCl, and 0.1% Tween 20) for 1 h. Blots were incubated in primary antibody solutions (1:1000) overnight and then washed and incubated in appropriate horseradish peroxidase-conjugated secondary antibody solutions (1:5000, Rockland) for 1 h the next day. Blots were probed with the following antibodies: anti-CCP1 (Proteintech, rabbit) and anti-glyceraldehyde 3-phosphate dehydrogenase (GAPDH; EMD Millipore, mouse), which served as a loading control. Bands were visualized using the LI-COR Biosciences digital imaging system, and pixel intensity was analyzed using ImageJ (NIH). CCP1 band intensities were normalized to corresponding GAPDH band intensities.

### Experimental design and statistical analyses

All statistical analyses were performed using a combination of GraphPad Prism (version 5.01, GraphPad Software) and Microsoft Excel (Version 14.0.7106, 32-Bit, Microsoft Corporation).

## Results

### Knock-down of CCP1 decreases neuronal survival of excitotoxic challenge

Loss of CCP1 has long been known to lead to severe neuronal degeneration in the murine CNS (Fernandez-Gonzalez et al., 2002). More recently, it was reported that loss of CCP1 in humans results in degeneration of cerebellar neurons and spinal cord neurons (Shashi et al., 2018). Therefore, we sought to test how CCP1 function affects the survival of spinal cord neurons.

**Figure 1.**
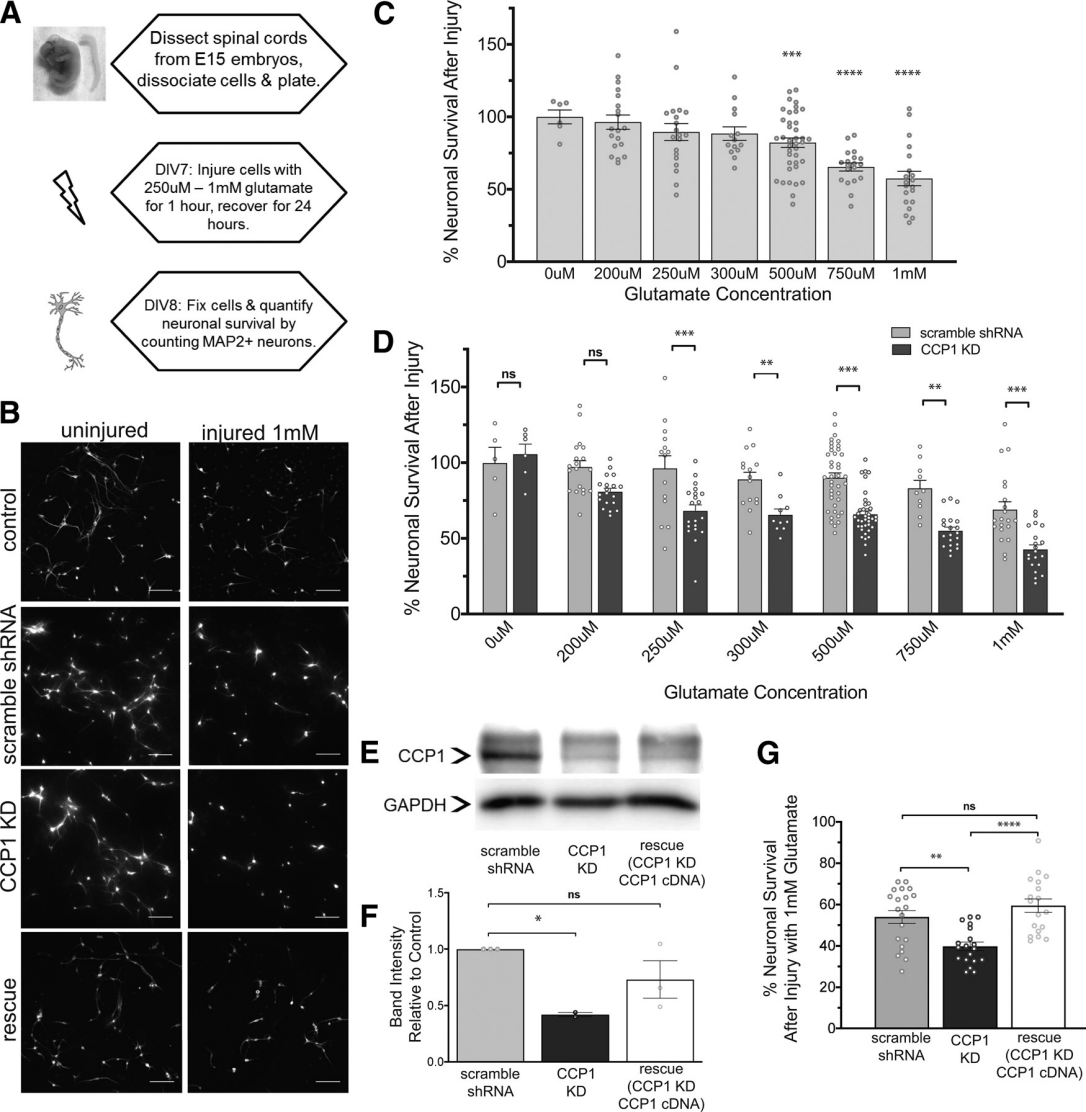
Knock-down of CCP1 decreases neuron survival in an *in vitro* model of glutamate-induced excitotoxicity. ***A***, Schematic of treatment of spinal cord cultures with excitotoxic concentrations of glutamate. Primary spinal cord cultures containing neurons and glia were treated with varying concentrations of glutamate for 1 h on DIV7 and allowed to recover for 24 h before fixation (see also Extended Data Fig. 1-1*A*,*B*). ***B***, Control and injured spinal cord cultures were immunostained for MAP2 to identify neurons. Glutamate-induced excitotoxic injury leads to loss of neurons and retracted neuronal processes compared with that of uninjured neurons. Also shown are cultures infected with a lentivirus expressing CCP1 shRNA knock-down, and cultures subjected to CCP1 knock-down and shRNA resistant CCP1 cDNA rescue, which ameliorates neuronal death and retraction of processes after injury. Scale bars: 100 μm (see also Extended Data Fig. 1-1*C*,*D*). ***C***, Percent neuronal survival after excitotoxic injury decreases with increasing glutamate concentration. Error bars represent SEM; ***p <* *0.001, ****p <* *0.0001 versus 0 μm glutamate as determined by one-way ANOVA and Dunnett’s multiple comparison test. ***D***, Quantification of percentage of neuronal survival following treatment with concentrations of glutamate ranging from 200 μm to 1 mm. At every concentration, CCP1 knock-down cultures show significantly reduced neuronal survival compared with those expressing scrambled shRNA control as determined by two-way ANOVA followed by Bonferroni *post hoc* test analysis; *p <* *0.05, **p <* *0.01, ***p <* *0.001 (percentage neuronal survival of scramble shRNA control groups was not significantly different from controls shown in ***C***). ***E***, Western blotting showing CCP1 in scramble, knock-down, and rescued cultures. GAPDH loading controls are also shown. ***F***, Quantification of Western blotting revealed that shRNA-resistant cDNA expression in CCP1 knock-down cells rescues CCP1 to levels similar to controls; *p *<* *0.05, determined by paired *t* test, *n* = 2. ***G***, Quantification of percentage neuronal survival treated with 1 mm glutamate. Neuronal survival of rescue cultures and control cultures are not significantly different as determined by one-way ANOVA followed by Tukey’s multiple comparison test; **p <* *0.01, ****p <* *0.0001. ns, not significant.

We isolated and dissociated embryonic rat spinal cords (Fig. 1*A*; Extended Data Fig. 1-1*A*) from pregnant mothers at E15 and grew mixed cultures of astrocytes, microglia, and neurons (Fig. 1*B*; Extended Data Fig. 1-1*B*). We subjected our cultures to glutamate-induced excitotoxicity, which is characteristic of the secondary phase of spinal cord injury (Du et al., 2007), by incubating the cultures with concentrations of glutamate ranging from 200 μm to 1 mm for 1 h on DIV7 (Fig. 1*C*). After injury, cultures were allowed to recover for 24 h before fixation on DIV8 and subsequent immunostaining for the neuronal marker MAP2. MAP2-immunopositive surviving neurons were counted, and as expected, glutamate exposure caused neuronal death in a dose-dependent manner (Fig. 1*B*,*C*). Approximately 60% of neurons survived following exposure to 1 mm glutamate for 1 h.

10.1523/ENEURO.0431-20.2021.f1-1 Extended Data Figure 1-1 Embryonic spinal cord cultures contain a mixture of neurons, astrocytes, and microglia at DIV7. ***A***, Image of a rat embryo collected at E15 (left) and a dissected spinal cord at this developmental stage (right). ***B***, Primary spinal cord cultures derived from embryos at E15 contain neurons (MAP2-immunopositive), astrocytes (GFAP+), and microglia (IBA1+) at DIV7, as labelled by immunocytochemistry. ***C***, shRNA-mediated knock-down of CCP1 affects neurons. Quantification of percentage of transduction by cell type. ***D***, Representative images of primary spinal cord cultures infected with a lentivirus carrying CCP1 shRNA. Neurons were immunostained for MAP2 (red), and infected neurons express GFP as a fluorescent transduction marker. Scale bars: 100 μm. Download Figure 1-1, EPS file.

To determine whether CCP1 is required for neuronal survival following glutamate-induced excitotoxicity, we used a lentivirus containing an shRNA sequence targeting the 3′ untranslated region (UTR) of CCP1 to knock down CCP1 expression. This sequence was found to be the most efficient at knocking down CCP1, as other viruses containing shRNA targeting the 5′ UTR or the coding region resulted in a lower transduction efficiency (data not shown). Cultures in which CCP1 levels were knocked down by lentiviral shRNA treatment were subjected to varying concentrations of glutamate. Neuronal survival following glutamate exposure was significantly reduced in cultures transduced with shRNA CCP1 knock-down at all glutamate concentrations tested (Fig. 1*A*,*B*,*D*).

To confirm that the knock-down of CCP1 is responsible for decreased neuronal survival following glutamate-induced excitotoxicity, we used Western blot analysis to show that CCP1 levels were reduced by ∼40–50% by shRNA expression (Fig. 1*E*,*F*). Additionally, we co-infected cultures with two lentiviruses carrying CCP1 shRNA and an shRNA-resistant CCP1 cDNA construct and found that CCP1 levels were rescued to levels similar to controls in these cultures (Fig. 1*E*,*F*). Cultures in which levels of CCP1 were rescued exhibited neuronal survival after excitotoxic challenge comparable to control cultures (Fig. 1*G*). As expected, the lentivirus containing shRNA specific to CCP1 selectively infected more MAP2-positive neurons than non-neuronal cells, such as glia (Extended Data Fig. 1-1*C*,*D*). Therefore, the shRNA-mediated decrease in CCP1 levels in these spinal cord cultures is most likely because of knock-down of CCP1 in neurons.

We conclude that the presence of CCP1 in neurons is necessary for protection from excitotoxicity.

### CCP1 levels decrease within 24 h after excitotoxic injury

**Figure 2.**
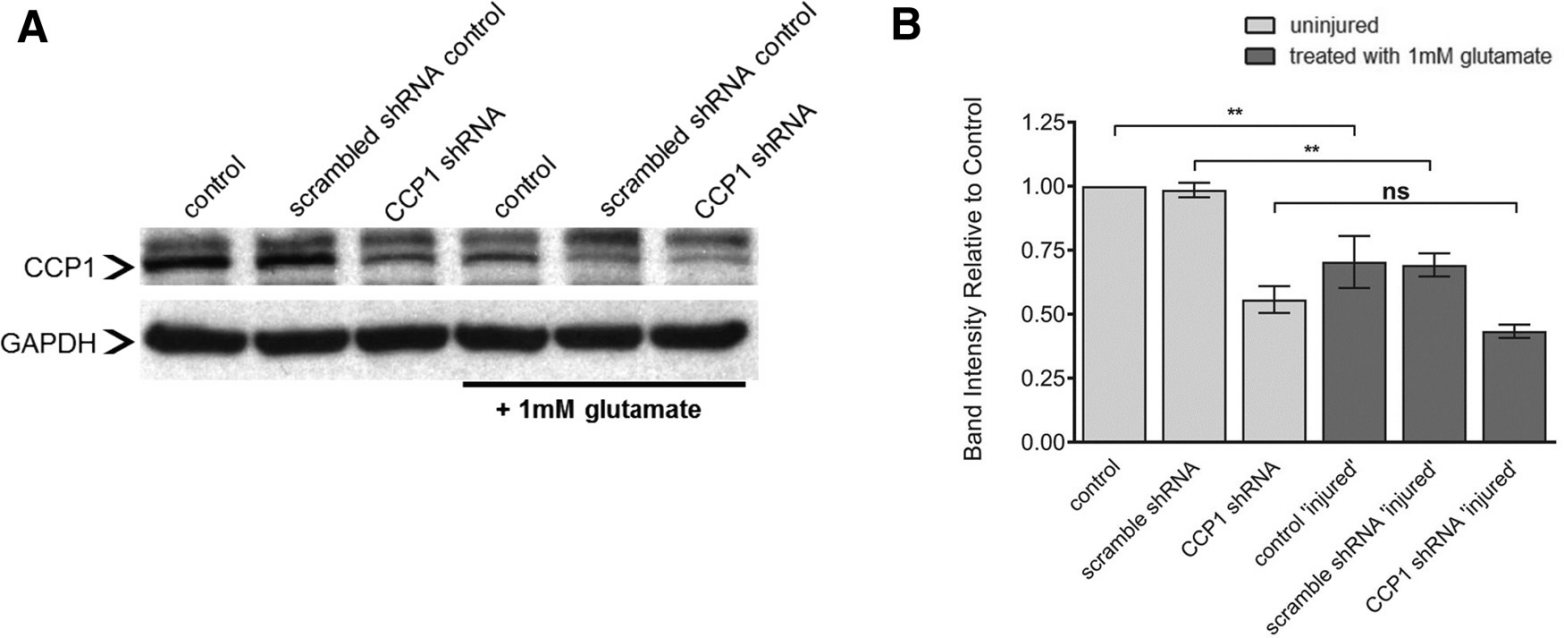
CCP1 is regulated after glutamate-induced excitotoxicity *in vitro.* ***A***, Western blotting showing changes in CCP1 before and at 24 h after injury. GAPDH was used as a loading control. ***B***, Quantification of CCP1 band intensity (mean ± SEM) from panel ***A***. All analyses determined by one-way ANOVA of relative band intensity followed by Tukey’s multiple comparison test; **p <* *0.01, *n* = 3. See also Extended Data Figure 2-1*A*. ns, not significant.

We next sought to determine whether glutamate-induced excitotoxicity affects protein levels of CCP1. Western blot analysis indicates that 24 h after excitotoxic injury, the level of CCP1 decreased to ∼60% of basal levels in uninjured cells (Fig. 2*A*,*B*; Extended Data Fig. 2-1*A*). Cultures treated with lentiviral CCP1 shRNA knock-down did not show an additional decrease in CCP1 levels after glutamate exposure. Because lentiviral infection was more efficient in neurons than in glial cells (Extended Data Fig. 1-1*C*,*D*), our results suggest that the levels of CCP1 protein are downregulated in neurons after excitotoxic injury.

10.1523/ENEURO.0431-20.2021.f2-1 Extended Data Figure 2-1 Results of binding of CCP1 antibody in Western blot assays and spinal cord cultures lack cholinergic motorneurons. ***A***, Arrow indicates CCP1 band at 137kDa, identifiable by size and pattern across control and CCP1 knock-down groups. Other bands did not change across groups and were considered non-specific cross-reacting proteins. ***B***, Representative images of MAP2 and ChAT immunostaining of mixed spinal cord cultures. Lack of ChAT immunostaining indicates that the neuronal population does not include motor neurons, and is most likely composed of interneurons. Scale bars: 100 μm. Download Figure 2-1, EPS file.

### Mammalian spinal cord interneurons in culture are ciliated

**Figure 3.**
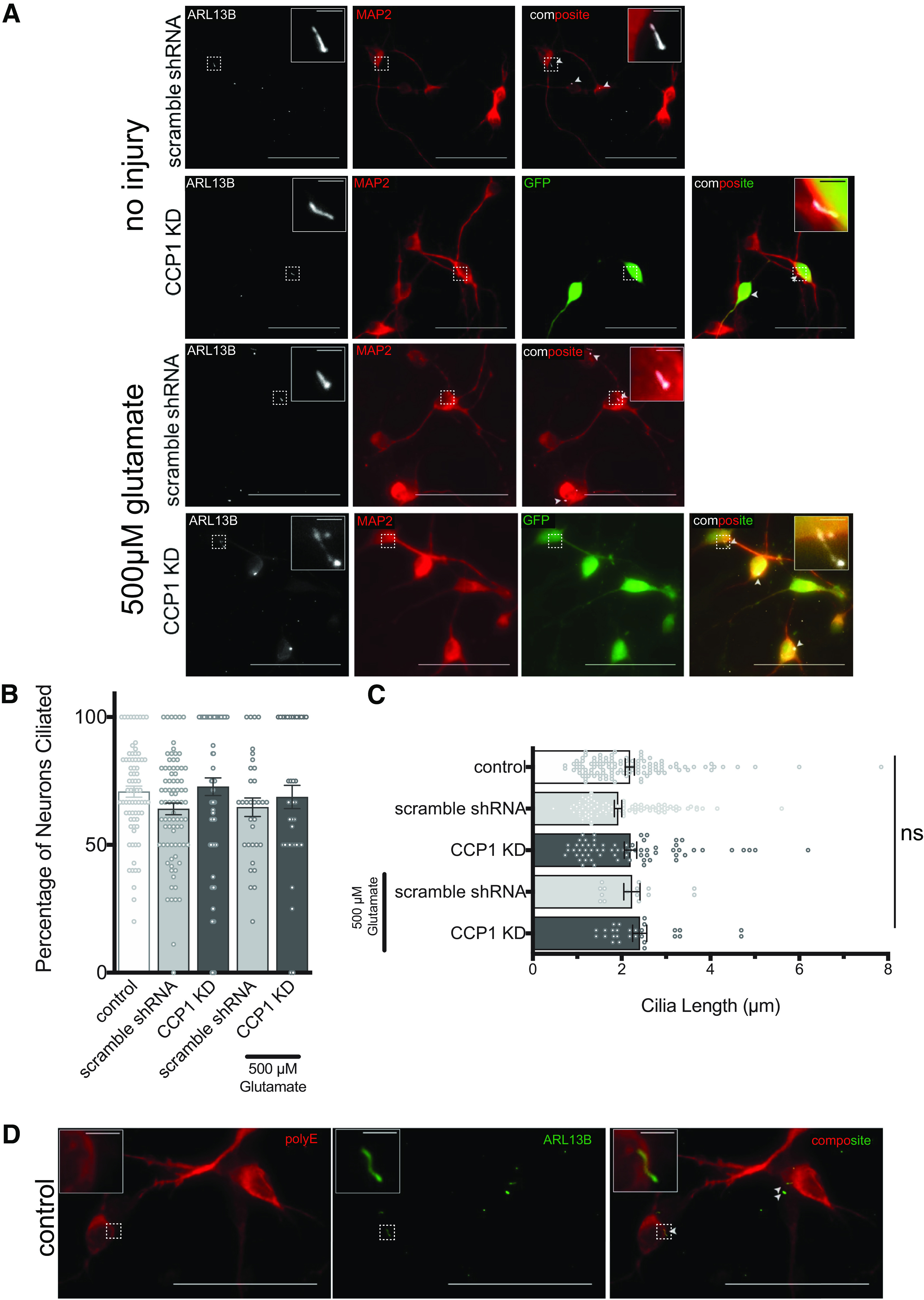
Interneurons in spinal cord cultures are ciliated. ***A***, Primary spinal cord cultures immunostained for MAP2 and ARL13B, showing ciliated uninjured neurons and ciliated neurons following treatment with 500 μm glutamate. Spinal cord cultures do not contain motorneurons (see also Extended Data Fig. 2-1*B*). ***B***, The percentage of neurons that are ciliated (mean ± SEM) show no significant differences with shRNA treatments or injury (ANOVA). ***C***, Average lengths of cilia (mean ± SEM) show no significant differences with shRNA treatments or injury (ANOVA). ***D***, Immunofluorescent detection of polyglutamylation (polyE) suggests that neuronal MTs, except those in cilia, are polyglutamylated in spinal cord cultures. No polyE immunostaining is observed in ARL13B-immunopositive cilia. Dotted line boxes indicate areas of insets. Scale bars: 50 and 2.5 μm. Arrowheads in composite views indicate cilia and inset areas are indicated by dotted line boxes. ns, not significant.

Primary cilia can play a neuroprotective role in the rodent CNS (Choi et al., 2019) and are important for reception of cellular signaling that is essential for development of the mammalian nervous system (Louvi and Grove, 2011; Bay and Caspary, 2012; Youn and Han, 2018). Furthermore, the loss of primary cilia causes neurodegeneration in mammals (Bowie and Goetz, 2020). Because CCP1 and glutamylation are implicated in ciliary degeneration and dysfunction, as well as neurodegeneration in rodents and humans (Campbell et al., 2002; Fernandez-Gonzalez et al., 2002; Chakrabarti et al., 2008; Kim et al., 2010; O’Hagan et al., 2011; Kubo et al., 2015; Branham et al., 2016; Power et al., 2020), we hypothesized that the neuroprotective effects of CCP1 arise from ciliation of spinal cord neurons. However, cilia of spinal cord neurons have not been well-documented. To our knowledge, only the CSF-contacting neurons (Djenoune et al., 2014; Orts-Del’Immagine et al., 2014; Böhm et al., 2016; Sternberg et al., 2018), neuronal precursors, and ependymal cells that line the central canal (Meletis et al., 2008) and motor neurons of the lumbar spinal cord (Ma et al., 2011) are ciliated. Therefore, we first assessed whether spinal cord neurons from cultured rat embryonic spinal cord are ciliated. Indirect immunofluorescence, using an antibody specific to the ciliary membrane protein ARL13B (Higginbotham et al., 2012), demonstrates that 57% of MAP2-immunostained spinal cord neurons in our cultures are ciliated at DIV8 (Fig. 3*A*,*B*). Because the dorsal root ganglia were removed from spinal cords before dissociating the cells, primary sensory neurons are not present in our embryonic spinal cord cultures. Interneurons are reported to make up ∼97% of all neurons in the spinal cord (Hochman, 2007). Moreover, motor neurons do not survive in embryonic spinal cord cultures under similar culture conditions (Kushima and Hatanaka, 1992), and no neurons in our cultures were positive for immunostaining for the cholinergic motor neuron marker ChAT (Extended Data Fig. 2-1*B*). Therefore, ciliated MAP2-positive cells in our cultures are interneurons.

### Knock-down of the deglutamylase CCP1 increases the presence of cilia on spinal cord neurons but does not significantly affect ciliary length

We hypothesized that if CCP1 functions to maintain cilia in mammalian spinal cord neurons, as it does in *C. elegans*, then loss of CCP1 function would cause ciliary degeneration (O’Hagan et al., 2011). We counted cilia present on neurons under baseline conditions and with CCP1 knocked down and measured their lengths. Surprisingly, we found that shRNA-mediated knock-down of CCP1 increases the frequency of ciliated neurons (Fig. 3*A*,*B*). However, neither CCP1 knock-down nor excitotoxic injury with 500 μm glutamate affected ciliary length (Fig. 3*C*). Thus, our data suggest that reduction of CCP1 does not lead to degeneration of primary cilia in the murine spinal cord, as it does in *C. elegans* sensory neurons, at least on a time scale of several days.

### Presence of neuronal cilia is not correlated with survival of excitotoxic injury

Cilia can play a neuroprotective role in the CNS as an antagonist to the cell cycle by inhibiting cell division and preventing apoptosis (Choi et al., 2019). To assess whether the presence of cilia plays a role in neuronal survival after glutamate-induced excitotoxicity, we compared the fraction of ciliated neurons after treatment with 500 μm glutamate to uninjured neurons. We hypothesized that if ciliation is neuroprotective, the frequency of cilia would increase in the neurons remaining after excitotoxic treatment, because of a decreased likelihood of survival of neurons without cilia. We found that in injured cultures, the percentage of neurons that were ciliated in CCP1 knock-down-treated cultures was ∼69% versus 64% in scramble shRNA-treated controls (Fig. 3*A*,*B*), a not statistically significant difference (Fig. 3*B*). The length of neuronal cilia was also not significantly different between CCP1 knock-down and scramble shRNA-treated glutamate-injured cultures (Fig. 3*C*). Therefore, our data suggest that cilia are not needed for CCP1 to protect sensory spinal cord neurons from excitotoxic death. Because CCP1 reduces the length of polyglutamylation side-chains on α and β tubulins (Rogowski et al., 2010), we tested whether the cilia that decorate spinal cord interneurons in our cultures have long polyglutamylation side-chains. We performed indirect immunofluorescence using a polyE antibody, which detects glutamate side-chains of three or more glutamates (Rogowski et al., 2010). The polyE antibody strongly immunostained neuronal cell bodies and neurites, but not ARL13B-positive cilia (Fig. 3*D*). Thus, ciliary MTs in spinal cord interneurons may have few or no polyglutamylation side-chains.

## Discussion

Several lines of evidence support the idea that glutamylation acts as a component of the tubulin code to regulate the MT cytoskeleton and MT-based motors. Glutamylation is a posttranslational modification found on stable MTs (Fukushima et al., 2009; Janke and Bulinski, 2011; Wloga et al., 2017). MT glutamylation regulates MT-based motor trafficking in neurons of *C. elegans* and mice (Ikegami et al., 2007; O’Hagan et al., 2011, 2017; Magiera et al., 2018) and in *in vitro* studies using purified tubulins and motors (Sirajuddin et al., 2014). Defects in tubulin modifications, MT stability, and neuronal trafficking are linked to neurodegenerative diseases, such as Parkinson’s, Huntington’s, and Alzheimer’s diseases (Baas et al., 2016; Matamoros and Baas, 2016; Brady and Morfini, 2017; Vu et al., 2017). Additionally, in humans, loss of the deglutamylase CCP1 (and resulting hyperglutamylation of MTs) leads to fatal infantile neurodegeneration in the spinal cord, cerebellum, and peripheral nerves (Shashi et al., 2018).

Neuronal expression of CCP1 (also known as NNA1 or AGTPBP1) is upregulated after injury of the sciatic nerve, and elevated expression is maintained during target reinnervation (Harris et al., 2000), suggesting that CCP1 plays a role in neuroregeneration and axonal regrowth after injury. Therefore, control of MT glutamylation could represent an important survival factor in the context of both neurodegenerative disease and neuronal injury. In order for regeneration after injury to occur, neurons must also survive (Dusart et al., 2005; Hollis et al., 2009). In this work, by combining lentivirally-delivered shRNA knock-down and a previously established *in vitro* model of the secondary phase of spinal cord injury (Du et al., 2007), we show that CCP1 promotes survival of neurons challenged with excitotoxic levels of glutamate.

We also hypothesized that CCP1 functions in cilia to promote survival of neurons exposed to excitotoxic injury. Cilia can play a neuroprotective role in the rodent CNS (Choi et al., 2019) and are important for reception of cellular signaling that is essential for development of the mammalian nervous system (Louvi and Grove, 2011; Bay and Caspary, 2012; Youn and Han, 2018). Loss of neuronal cilia, caused by mutation of TTBK2 or by conditional knock-out of the ciliary intraflagellar transport protein Ift88, causes degeneration of Purkinje neurons of the cerebellum (Bowie and Goetz, 2020). Motile cilia on ependymal cells, such as those that line the central canal, are essential for spinal cord morphogenesis (Grimes et al., 2016). Cerebrospinal fluid-contacting neurons, which also have a motile cilium, extend an apical projection into the central canal and are proposed to relay cerebrospinal fluid flow and pH information spinal circuits for normal development and function of spinal cord nerves (Böhm et al., 2016; Djenoune et al., 2017; Sternberg et al., 2018). Spinal cord injury can cause degeneration of motile ependymal cilia or ependymal cells, which create cerebrospinal fluid flow in the central canal and neurons (Radojicic et al., 2007). This could result in toxic buildup of CNS waste, possibly preventing regrowth and exacerbating the chronic degeneration of spinal tissues after SCI (Radojicic et al., 2007). CCP1 homologs have been found to regulate the integrity and structure of MTs in cilia, the function of ciliary motors (Suryavanshi et al., 2010; O’Hagan et al., 2011, 2017; Kubo et al., 2015; Hong et al., 2018), and ciliary length (Kim et al., 2010).

To our knowledge, no previous reports have demonstrated that spinal cord interneurons are ciliated. Spinal cord motorneurons have been shown to be ciliated *in vitro* (Ma et al., 2011). We used immunofluorescence to detect the ciliary protein ARL13B and found that most neurons in embryonic spinal cord cultures are ciliated. Importantly, under the culture conditions we used, spinal cord cultures lack sensory neurons and motorneurons (Extended Data Fig. 2-1*B*; Kushima and Hatanaka, 1992). Therefore, our data provide the first evidence (to our knowledge) that spinal cord interneurons are ciliated.

Our data suggest that expression of CCP1 may not strongly regulate ciliation in spinal cord neurons. This was surprising, as we had previously demonstrated that the lack of a CCP1 homolog in nematodes causes the degeneration ciliary MTs and of neuronal cilia (O’Hagan et al., 2011). An siRNA screen in immortalized human retinal pigmented epithelial cells had also found that CCP1 can positively regulate cilia length (Kim et al., 2010). Our result may be explained by the fact that the tubulin code, and glutamylation in particular, can result in cell-specific effects (O’Hagan et al., 2011, 2017), likely because of differences in expression of genes that read, write, or interpret the tubulin code modifications. Even within a single cell, different populations of MTs could have different glutamylation states, endowing them with specific properties. The fact that loss of CCP1 function in humans, mice, and sheep results in degeneration of particular neurons, such as cerebellar Purkinje cells and spinal cord motor neurons (Fernandez-Gonzalez et al., 2002; Zhao et al., 2012; Shashi et al., 2018), rather than all neurons, may reflect such differences in glutamylation in different neuronal types. In our cultures, we observed that the polyE antibody detected polyglutamylation throughout neurons but not in cilia. This could explain why CCP1-knock-down reduced neuronal survival but did not result in degeneration of neuronal cilia in our cultures: ciliary MTs with minimal polyglutamylation might not require activity of CCP1, which functions to reduce the length of glutamate side-chains. Therefore, CCP1 might affect neuronal survival by reducing polyglutamylation on MTs in compartments other than cilia, such as axons or dendrites. However, using our *in vitro* system, we cannot address how CCP1 might function in cilia of ependymal cells, cerebrospinal fluid-contacting neurons, or motor neurons in response to nerve injury.

Because CCP1 reduces the length of polyglutamylation side-chains on MTs (Rogowski et al., 2010) and polyglutamylation regulates MT severing (Janke and Magiera, 2020), we envision that regulation of CCP1 expression and function is necessary to appropriately reorganize MT dynamics following neuronal injury to facilitate axonal regeneration and recovery. This notion is supported by the previous finding that CCP1 homologs in the invertebrate *C. elegans* are necessary for normal neuronal outgrowth following axotomy of touch receptor neurons (Ghosh-Roy et al., 2012). When CNS axons fail to regenerate, MTs are disorganized after injury, whereas regenerating PNS axons form organized MT networks in their growth cones (Ertürk et al., 2007), suggesting that one difference in the regenerative potential of CNS versus PNS neurons is the capability of reorganizing the cytoskeleton for regrowth. Additionally, MT defects are proposed to underlie neurodegeneration after traumatic brain injury (Tang-Schomer et al., 2010).

Our results suggest that CCP1 function is needed to improve neuronal survival of excitotoxic injury. We found that CCP1 expression significantly decreased following glutamate-induced excitotoxicity in spinal cord neurons. When CCP1 was knocked down before injury, excitotoxicity did not further reduce CCP1 expression. Our observations differ from a previous study (Harris et al., 2000), which reported upregulation of CCP1 following transection or nerve crush injury of the sciatic nerve in the PNS. There are at least two possible explanations for the observed difference in CCP1 levels. Acute injury that severs or damages the axonal cytoskeleton might increase CCP1 expression, while chronic (excitotoxic) injury might decrease CCP1 expression. Alternatively, injury of PNS neurons might upregulate CCP1 expression, while injury of the CNS might downregulate of CCP1 expression. In this case, differences in the function or structure of the MT cytoskeleton, mediated at least in part by CCP1, might explain the poor regenerative potential in the CNS and robust regeneration in the PNS.

Pharmacological targeting of tubulin-modifying enzymes has been proposed as a new approach to treating neurodegenerative disease (Rogowski et al., 2021). We suggest that drugs that target enzyme regulators of posttranslational MT modifications might reduce cumulative neuronal damage from excitotoxicity during the secondary phase of CNS injury. Our data support the idea that direct or indirect activation of CCP1, or prevention of its downregulation by excitotoxicity, might protect injured neurons. Future investigations could include methods to inactivate TTLL glutamylase enzymes that oppose CCP1 by initiating or elongating polyglutamylation side-chains (van Dijk et al., 2007; Mahalingan et al., 2020), which might also provide a neuroprotective effect by reducing glutamylation of MTs.

## Synthesis

Reviewing Editor: Eran Perlson, Tel Aviv University Decisions are customarily a result of the Reviewing Editor and the peer reviewers coming together and discussing their recommendations until a consensus is reached. When revisions are invited, a fact-based synthesis statement explaining their decision and outlining what is needed to prepare a revision will be listed below. The following reviewer(s) agreed to reveal their identity: Mónica Sousa.

The authors answer my concerns
